# The Challenges of Working in the Heat Whilst Pregnant: Insights From Gambian Women Farmers in the Face of Climate Change

**DOI:** 10.3389/fpubh.2022.785254

**Published:** 2022-02-10

**Authors:** Shantelle Spencer, Tida Samateh, Katharina Wabnitz, Susannah Mayhew, Haddijatou Allen, Ana Bonell

**Affiliations:** ^1^Medical Research Council Unit, The Gambia at London School of Hygiene and Tropical Medicine, Banjul, Gambia; ^2^Chair for Public Health and Health Services Research, Institute for Medical Information Processing, Biometry and Epidemiology (IBE), Ludwig-Maximilians-Universität München, Munich, Germany; ^3^London School of Hygiene and Tropical Medicine, Faculty of Public Health and Policy, University of London, London, United Kingdom; ^4^Centre on Climate Change and Planetary Health, London School of Hygiene and Tropical Medicine, University of London, London, United Kingdom

**Keywords:** climate change, health, women, climate adaptation, The Gambia, occupational heat stress

## Abstract

**Background:**

The expected increase in heat in The Gambia is one of the most significant health threats caused by climate change. However, little is known about the gendered dynamics of exposure and response to heat stress, including women's perceived health risks, their adaptation strategies to heat, and their perceptions of climate change. This research project aims to answer the question of whether and how pregnant farmers in The Gambia perceive and act upon occupational heat stress and its health impacts on both themselves and their unborn children, against the backdrop of current and expected climatic changes.

**Method:**

In-depth semi-structured interviews were conducted with 12 women who practice subsistence farming and were either pregnant or had delivered within the past month in West Kiang, The Gambia. Participants were selected using purposive sampling. Translated interview transcripts were coded and qualitative thematic content analysis with an intersectional lens was used to arrive at the results.

**Results:**

All women who participated in the study experience significant heat stress while working outdoors during pregnancy, with symptoms often including headache, dizziness, nausea, and chills. The most common adaptive techniques included resting in the shade while working, completing their work in multiple shorter time increments, taking medicine to reduce symptoms like headache, using water to cool down, and reducing the amount of area they cultivate. Layered identities, experiences, and household power structures related to age, migration, marital situation, socioeconomic status, and supportive social relationships shaped the extent to which women were able to prevent and reduce the effects of heat exposure during their work whilst pregnant. Women who participated in this study demonstrated high awareness of climate change and offered important insights into potential values, priorities, and mechanisms to enable effective adaptation.

**Conclusion:**

Our findings reveal many intersecting social and economic factors that shape the space within which women can make decisions and take adaptive action to reduce the impact of heat during their pregnancy. To improve the health of pregnant working women exposed to heat, these intersectionalities must be considered when supporting women to adapt their working practices and cope with heat stress.

## Introduction

Climate change is expected to increase the frequency of extreme heat events and change precipitation patterns globally (1). These changes multiply health threats and amplify the health inequities of the most at-risk populations and individuals, and women, and in particular pregnant women, are amongst the groups most directly threatened by climate change (2).

In West Africa, climate change is expected to reduce crop production, compromising the food security of subsistence farmers, and rendering livelihoods based on natural resources more challenging, particularly in the Sahel zone (3). Recent studies in West Africa have shown that differences in age, gender, education, disability as well as livelihood diversification significantly influence levels of vulnerability to the effects of climate change (4). Under the current warming scenarios of 1.5°C to 2°C, about 50 to 100% of the population in most of the Sahel countries are expected to be at risk of the most dangerous effects of heat (5).

The expected increase in heat is one of the most significant health threats caused by climate change. Exposure to both high temperature and humidity can lead to occupational heat stress that can substantially affect people who work outdoors in hot environments (5–7). This will be an important challenge faced by smallholder farmers in The Gambia, where most agriculture is rain-fed, non-mechanized, and the timing of the agricultural seasons requires farmers to work year-round in temperatures exceeding 30°C (8). Strict cultural customs and gender norms limit the land available to and the crops accessible to be cultivated by men and women, reflecting social hierarchies of gender, marital status, and age.

Few studies have laid a particular focus on women or women farmers and their experience of climate change, as well as their agency in climate change adaptation. Women's voices are often missing from climate change mitigation and adaptation discussions and policy decision-making processes (9), a critical gap recognized by the international community (8). Alternatively, women are portrayed as passive victims of climate change processes beyond their awareness or control (10), further exacerbating gender inequalities in the impact of climate change and excluding this group capable of acting as powerful environmental protectors (11). Although the Gambian government has made firm and far-reaching commitments to ensure the country is on track to meet the Paris Climate Accords, a recent interim report evaluating the impact of the UN environment programme project found that there was a general failure to include women into decision making roles, despite this being a stated aim (8). Therefore although women constitute around 50% of the agricultural workforce in The Gambia, their involvement in decision-making and their access to assets are limited, and they have been shown to be more vulnerable to climate change due to their dual productive and reproductive role (12).

Globally, few studies focus on the gendered dynamics of exposure and response to heat stress, including women's perceived health risks and their adaptation strategies to heat (13). Epidemiological datasets on the effects of heat on pregnant women in Africa are scarce, though there is evidence from other settings that there is an increased risk of adverse birth outcomes attributable to heat exposure (14–17). Associations between temperature and birth outcomes are largest among women in lower socioeconomic groups and at age extremes (18), revealing important aspects of identity beyond gender that influence susceptibility to heat stress.

When pregnant, Gambian women generally do not enjoy privileges in their households, with the division of labor in the household requiring women, albeit pregnant, to endure heavy workloads with limited opportunities for sick leave, and often to remain engaged in non-remunerable field work with few economic resources (19). The implications of these factors on the ability of Gambian women subsistence farmers to adapt to increasing temperatures brought on by climate change, during their pregnancy and otherwise, merits further exploration and representation in the climate change discourse. Furthermore, as far as we are aware, no effort has been undertaken to investigate the understanding and experiences of female farmers in The Gambia around the health impacts of heat and their personal adaptation strategies surrounding pregnancy and reproductive health.

This research project is set out to answer the question whether and how women in The Gambia, who depend mainly on subsistence farming and who are either pregnant or have delivered recently, perceive and act upon occupational heat stress and its health impacts on both themselves and their unborn children against the backdrop of current and expected climatic changes. As such, this study was designed to contribute toward the development of a contextualized understanding of how climate change is affecting the health of female subsistence farmers within The Gambia during pregnancy, explore the layered social factors that shape their adaptation strategies, and begin to center the most affected individuals in the design of effective and inclusive climate change adaptation strategies.

This study complements the observational study on heat stress and fetal well-being undertaken in Keneba, The Gambia (20). The aligned outcomes of both projects will help to inform future research as well as inclusive and community-centered health promotion strategies. The knowledge gained will also help to inform future research to understand the nuances of climate change perceptions and adaptive capacity, and inform potential programmatic or policy interventions on adaptation to climate change in West Africa.

## Methods

### Theoretical Framework

Much of climate change literature adopts a vulnerability and adaptation framework (21), and often individuals experiencing the effects of climate change are conceptualized as passive victims (22). Similarly, studies that discuss gender in relation to climate change often give disproportionate emphasis on the implications of climate change using a men vs. women dichotomy, with little attention paid to power and social relations among varied identities within these groups (21). This study aims to align with Djoudi's call for an intersectional perspective of climate change that recognizes individual agency and creates spaces for emancipatory change (21).

As such, in this study we situate our approach and analysis within intersectional theory. The concept of intersectionality can address some of the important issues in the debates on vulnerability and adaptive capacity in the face of climate change, as it illuminates how different identities relate to climate change in relation to context specific power structures (9). Grounded in Black feminist theory in the US and first developed as a critical theory by Crenshaw in the early 1990's, intersectional theory recognizes that individuals carry multiple, intersecting identities and privileges associated with these intersections, including ethnicity, race, gender, sexual orientation, social class, and disability (23). Intersectional theory offers an analytical approach to understanding and examining the interconnectedness of numerous socially constructed identities (e.g., race, gender, sexual orientation, class, etc.) as they collectively shape the lived experiences of individuals and groups (24). This theoretical approach requires moving beyond binaries to recognize the complex ways in which power and systems intersect with aspects of identity to shape the lived experience of people, enabling an analysis that reflects the complexity of social issues and outcomes (25). Intersectionality can be used to generate critical and constructive insights, critique existing power relations and institutional practices, and generate alternative knowledge crucial in the formulation of more effective and legitimate climate strategies (9).

In the context of this study, an intersectional theoretical framework allows reflection and analysis on the many intersecting factors that shape women's experience of climate change, heat stress, and reproductive health, and explore how gender norms, class, family structure, displacement, and disability affect levels of exposure to heat, ones' ability to implement adaptive changes during pregnancy, as well as the consequences of adaptive strategies on personal and household wellbeing. To remain true to the critical focus of intersectional theory, a view of intersectionality must also acknowledge the deep need for social transformation, maintain an awareness of privileges and oppressions, and commit to a broad sense of justice (26). Therefore, this study aims to take an initial step toward eliciting the experiences and opinions of Gambian women farmers in order to center them more in the current discourse surrounding the effects of climate change and adaptation processes in The Gambia and globally, and to begin to inform policy approaches to climate change and extreme heat adaptation.

The application of intersectional theory also encourages critical self-reflexivity with regards to the ways in which power imbalances in researcher and participant identities can influence the process of qualitative inquiry (24). To this end, this study has brought together a diverse research team, consisting of Gambian and international researchers with contextual knowledge of The Gambia and lived experience in West Kiang as an attempt to balance potential hierarchies of power between the researchers and research participants during the research process. The research team represents a variety of cultural and academic backgrounds, as well as a range of experience with research in gender, fertility, heat physiology, and climate change adaptation in West Africa.

### Study Setting

**Figure 1:**
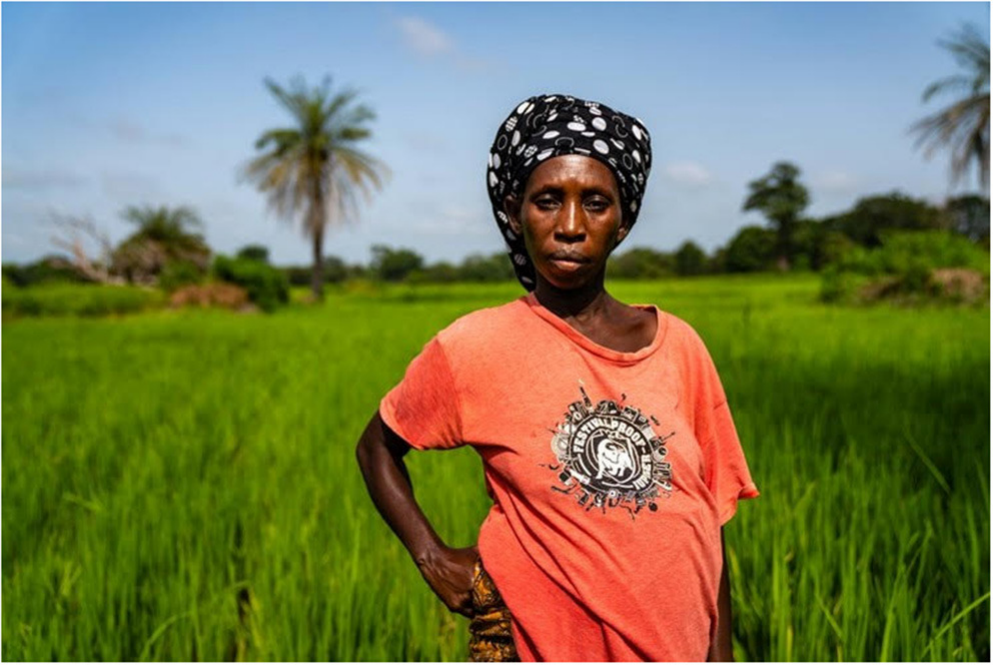
Binta is a woman residing in Keneba village, working in her rice fields while pregnant.

This study was conducted at Keneba field station, MRCG@LSHTM. This rural field station is 2.5–3 h from the coast and covers the West Kiang region which is an area of 36 villages with ~15,000 inhabitants where mostly subsistence farming is practiced. The climate in this area has two distinct seasons, a short wet season from June to October and a longer dry season from November to May. Farming of rice and groundnuts occurs during the wet season and farming of vegetables occurs during the dry season. Farming is gender specific, with women cultivating rice on communal plots during the rainy season, see Figure 1 and vegetables in communal plots during the dry season. Plots for cultivating rice belong to the family and are passed from mother to daughter. The vegetable plots are established communally, but each woman receives a certain number of beds to cultivate each season, from which they can sell or use the harvest for household subsistence. Men in turn cultivate cash crops to be sold or exported, such as groundnut, on plots of land owned by a household. All farming work is done manually, including watering of vegetable crops during the dry season. This requires farmers to carry buckets of water from hand-dug wells or boreholes.

Recruitment was focused on five villages selected due to their proximity (within 10 kms) of the Keneba MRCG@LSHTM Field Station. A list of currently pregnant women residing in these villages was generated from MRC antenatal clinic and DHS records. Women who performed outdoor labor in the form of rice farming or vegetable farming and were either pregnant or had been pregnant in the last six months met the inclusion criteria. Purposive sampling was then used to ensure the sample was diverse in terms of age and came from a range of communities, as well as including women who had had multiple pregnancies and those in their first pregnancy. All women on this list were invited to participate in the study by a fieldworker who resides in the area. Before the interviews, a Gambian researcher familiar to the community held an in-depth conversation with the potential participants to ensure their understanding of the purpose and methodology of the study, and their willingness to participate. All participants gave their written consent to participate in the study. Ethics approval was obtained through the Gambia Government/MRC joint ethics committee, and by the LSHTM ethics committee.

Between two and four interviews were conducted each day, and at the end of each day the interview notes were reviewed to examine emerging themes and plan further recruitment with the aim of reaching data saturation. After seven interviews were conducted, an additional eight women were approached to participate in the study, taking extra care to ensure representation of a range of ages and pregnancy experiences within the sample. Recruitment ended once thematic saturation was suspected, and the sample was reflective of a range of women of different ages and experiences. In all, 15 women were approached to participate in the study. Of these, 12 met the eligibility criteria of being pregnant or recently pregnant and actively engaged in farming during their pregnancy and were included in the study.

In-depth semi-structured interviews were conducted with 12 women who practice subsistence farming and were either pregnant or had delivered within the past month. Interviews sought to examine the ways in which women farmers who work outside during their pregnancies are exposed to heat, the physical effects of working in the heat whilst pregnant, the dynamics that shape how and whether women are exposed to heat, and what mechanisms and resources women draw upon to cope with the heat. The interviews also sought to understand the ways in which women view and are affected by climate change. Interviews were conducted by two researchers, with simultaneous translation from Mandinka to English conducted by one researcher during the interview. Interviewees were encouraged to share their perceptions and allow their ideas to unfold freely, sharing a nuanced account of their opinions and experiences. Drawing on intersectionality theory, follow-up questions and prompts were used to obtain clarity and deepen understanding on the ways in which intersecting identities, and the power and systems that surround them, shape women's experiences, as well as to gather nuanced insights and perspectives on recurring themes and topics. Interviews were conducted near the homes of the participants in a comfortable location that would provide privacy. Given the COVID-19 pandemic, all interviews were conducted outside, physically distanced by 2 m and all people present were provided with face masks. Interviews were recorded using two separate handheld voice recorders. Each interview lasted between 30 and 50 min. Following the interviews, pseudonyms were assigned to all participants to anonymize transcripts and ensure confidentiality. Transcription was completed by a researcher listening to the translation of the recordings. A sample of three interviews were reviewed by a Mandinka-speaking research assistant to verify accuracy of the translation and transcription.

Data analysis was conducted using thematic analysis, which aims to provide a summary of the regularities and variations within and across individual accounts (27). It is also suitable for exploring participants' worldviews and concepts inductively and iteratively. Starting with two initial themes linked to the objective of the study—experience of heat during pregnancy, and perceptions of climate change—patterns of recurring sub-themes and ideas within the data were noted and a coding scheme developed using NVivo-software.

After coding, common themes and the relationships between them were further grouped into specific themes, as represented in Table 1.

**Table 1:** Research topics and themes.

**Topic**	**Themes**
Experience of heat during pregnancy	Physical symptoms of heat during pregnancy
	Occupational heat stress during pregnancy
	Sources of support
	Personal coping mechanisms
Perceptions and understanding of climate change	Observed changes in weather and/or climate
	Gendered implications of climate change

## Results

### Characteristics of Research Participants

The 12 women who participated in this study were residing in one of the five selected villages in West Kiang, were currently or recently pregnant, and their primary occupation was farming. Of the 12, two had delivered their babies in the 10 days prior to the interview, and the remainder were between 4 and 9 months pregnant. The average age of participants was 32 years. Demographic information can be found in Table 2.

**Table 2:** Demography of 12 study participants.

**No**.	**Community**	**Age**	**Gender**	**Primary occupation**	**Number of children**
1	Community 1	22	F	Farming	0
2	Community 2	28	F	Farming/ selling food	9
3	Community 2	31	F	Farming	5
4	Community 2	32	F	Farming	7
5	Community 3	41	F	Farming/selling at shop	7
6	Community 3	45	F	Farming/selling ice	4
7	Community 4	40	F	Farming	7
8	Community 4	24	F	Farming/selling food	5
9	Community 4	29	F	Farming	4
10	Community 5	31	F	Farming	6
11	Community 5	30	F	Farming	3
12	Community 5	27	F	Farming	3

### Heat and Pregnancy

All participants in the study discussed their experience of a range of significant physical changes during pregnancy. Common symptoms included nausea, vomiting, fatigue, and dizziness. Nearly all women interviewed noted changes in their feelings toward their work during their pregnancies due to these physical symptoms. Most women discussed a decreased ability to do the amount of work compared to when not pregnant, due to increased feelings of tiredness or weakness brought on by their pregnancy. Many women noted that these symptoms were made worse when working in the heat. A few shared that there were no changes to how they felt about work at all when pregnant.

“*When you are not pregnant you can work very easily, very quick. When you are pregnant, even if you want to do it, you feel lazy, and easily tired.”—Ajara, age 29*

Some were more tired than usual, which rendered their work more challenging, and others noted some tasks becoming more difficult when pregnant, like bending and watering, especially during the dry season.

“*The work during the dry season, the work is harder than in the rainy season. During the rainy season only hard work you do in the rainy season is plowing, but in the dry season if you don't have water it will be difficult for you, you must have water for the beds…during the rainy season it is harder [to be pregnant], because you always bend, all the work you do you must bend.”—Zainabou, age 40*

Many noted differences in their experience with different pregnancies throughout their lives, indicating that some pregnancies had a more significant impact on their body, with physical symptoms increasing in severity with the number of pregnancies and their increasing age.

“*Each pregnancy is different, sometimes they are harder than others....All my previous pregnancies, I've felt normal. Sometimes I felt off. But this pregnancy, I don't usually get sick and lie down and do nothing, but I haven't been able to do my work like I usually want to. I heard people saying that when you have many children, more than 6, around 9, some of your last pregnancies you might feel uncomfortable, or encounter a lot of problems”—Isatu, age 32*

Several of the older women interviewed shared that they were hoping that this pregnancy would be their last—this was often due to the physical stress of working while pregnant when older in age, a stress in many cases aggravated by heat. Two women who had recently had tightly spaced pregnancies shared that they were interested in preventing future pregnancies completely, or spacing their pregnancies more to allow their body to rest, as pregnancy and birth was becoming increasingly challenging as they had more children.

“*Tiredness of work doesn't worry me, I am old now to have more children. Now I don't want to get pregnant unless God wills. The way it was difficult for me to bear this child, I've never experienced that in pregnancy to have a child before.”—Fatou, age 28*

### Occupational Heat Stress During Pregnancy

Women shared that they completed a range of different types of work throughout the day, with their primary occupation as farmers balanced among many unpaid care responsibilities, including caring for children and elderly family members, cleaning, doing laundry, fetching water, and cooking for the household. The tasks are spread throughout the day, with little time for rest or leisure in between. Gardening is often done in the mid-morning and early evening, after cooking breakfast and cleaning the compound, and before preparing lunch and dinner. Cultivating rice during the rainy season is usually done for the full day.

All women who participated in the study shared their experiences of considerable physical heat stress in their work as farmers and emphasized that their experience of heat stress is significantly more severe during pregnancy. The degree of heat stress varies throughout the year according to workload and exposure to heat. Most women reported their most important exposure to heat as occurring during the dry season, when the sun is most intense, but often exposure to heat was high during the rainy season as well, when humidity is higher and longer days are spent working in the rice fields. One woman shared:

“*When you work in the garden, you go and come back. When you are working in the rice field, you go, and you are there until evening.”*—*Ajara, age 29*

Women frequently noted a significant difference in their body's reaction to heat when they are pregnant, compared to when they are not pregnant. Many reported feeling the heat more acutely when pregnant, as well as a reduced ability to cool their bodies. Common symptoms were headache, dizziness, nausea, and chills. Interestingly there were no concerns regarding access to drinking water, or maintaining an adequate level of hydration throughout the day, either at the gardens or the rice fields.

“*When it is very hot, whenever I go out I feel dizzy, headache. When I'm not pregnant I don't feel the heat of the sun very much, but when I am pregnant, whenever I am out, I feel like my body is burning. I don't know for other ladies, but for me, I always feel hot inside my body, I always ask the children to pour water on me so that I can feel a little less warm.”—Aminata, age 45*

### Sources of Support

Supportive social relationships were frequently mentioned as important factors that enabled women to protect themselves from the heat. In some cases, women who worked with others around them would receive help with particularly challenging tasks during their pregnancy, such as watering or bending. Women with older children capable of helping with farming tasks shared that their children would often help with tasks like watering. However, this was not universal—often children were unable to help as they were going to school or occupied during working hours in the garden or the rice fields, or the woman did not feel comfortable asking those around her for help. Similarly, in some households a strictly gendered division of labor meant that male children would go only to help their father with cultivating cash crops, so a woman without daughters would be left to work alone in the garden or the rice fields.

“*My daughter is going to school and she is staying there, so my son is the only one who helps me at home but he goes to school as well, so he can only help when the school is closed.”—Aisha, age 30*

Having a mother, sister, or co-wife nearby was often stated as an important source of support for women during their pregnancy. Women often referred to their mothers or sisters as being the ones who would help them with their farming and domestic work if they were ill or unable to work. Another woman shared about receiving support from her sister's daughter during her pregnancies:

“*With my previous pregnancies I was staying with my sister's daughter, and she would help me with my domestic work. But for this one I am alone here.”*—*Aminata, age 45*

Two women shared about being able to divide household tasks between co-wives residing in the same household. They would alternate cooking shifts, allowing each of them a restful afternoon several times a week. Leaving one's village for marriage was often a factor that removed access to these supportive relationships, and often they were not replaced. A woman who had moved to a village for marriage shared that she felt uncomfortable asking people in her village for help with the garden, and experienced limited support from her partner with her work:

“*These people are not my relatives. My husband doesn't help me at the garden, he only helps me with the domestic work at home.”—Fatou, age 28*

Working in group settings was often mentioned as another important source of support. Women's plots are usually located within a larger group garden—each person cultivates their own plots, but their neighbors cultivate plots adjacent to theirs, and they share the same water access point. If sick for a few days, some women mentioned the women cultivating plots next to theirs, or their shift-mates would cover for them. In some cases, doing work collectively was not protective from the heat as it prevented their ability to self-pace their work, and take breaks when needed—one woman reported some social pressures around work from family members who work together, and having more freedom to make autonomous decisions when working individually.

“*In the rainy season, the work is more difficult than in the dry season, because in the rainy season you can't leave your work because you are working with other people. In the dry season during garden work, you separate from your mom and others. If you have your own work, you can leave your work and do what you can do, and nobody will say it is not fair that you are not working”—Isatu, age 32*.

The socioeconomic status of a household was an important factor that shaped the ability of women to implement coping strategies that involved reducing their workload or minimizing their exposure to heat by not working as intensively during their pregnancy. In some cases, their husband obtained a job, and as their children grew up they were able to help them by working in the field or by sending money from employment they had secured.

“*For my second pregnancy, when we used to work at the rice fields, and we would go there early in the morning to harvest the rice, we would have to use that rice for the day's lunch, we would pound it there to eat that day. That [amount of work] made me have low blood pressure…Then, our kids were very young, they were all going to school, and our husband wasn't working. Now, my husband is working, and our kids have completed schooling, and some are now working, so they support us. So now, we cultivate rice but it is minimized now, we don't need to harvest and use it that very day to eat, we just harvest and pack it”—Nyima, age 41*

### Personal Coping Mechanisms for Heat Stress During Pregnancy

Women shared a range of means that they used to reduce the effects of heat on their body while working outdoors during their pregnancy. Many of these involved taking additional time to rest while working in the field, drinking water, or using water to cool down. Others shared that they used ice to cool themselves down, or bathed before and after working:

“*Before, I would go with my water to the bush, park my water in the shade, and when it is hot, and I notice I'm tired and hot, I will use that water and drink, and sit under that tree for a while. In that time, I realize that the baby is free and comfortable, that's the time I will go and continue with my work.”—Aisha, age 30*.

“*I always take bath, and my sister at the coast, she sends ice from the coast for me to sell. When I have that ice, I take an ice block, and I use it all day. If I don't have that, then there are people who have ice, I will go there to have cool water to drink.”—Aminata, age 45*

Some women also shared that they would adapt their domestic work after working in the field, either shifting some tasks to the next day, or doing tasks in smaller batches or with other people to avoid straining.

“*When I close from work, when it's hot, I normally sit at one place, and ask my children to bring things to me. I also pass things to the next day, and just leave the work.”—Fatou, age 28*

One woman shared that she used medicine to help her with her headache, trying both paracetamol and herbal medicine to help with the side-effects of working in the heat.

“*I usually pour water on my head, when I have medication [tablets] with me I take it for the headache, and I relax for a while. When I take this medicine, and it doesn't help me, I also use herbs, I take them from the bush.”—Zainabou, age 40*

Many women shared that they must reduce their workload to be able to carry on working in the heat, even if they want to continue working as usual. When pregnant, some women will reduce the amount of beds they cultivate, leaving the other fields fallow, or offering them to a neighbor to cultivate instead.

“*When the sun is really hot, it makes you feel tired, and dizzy. The amount of work that you want to do usually you cannot do, your body will be heavy and you will feel dizzy.”*—*Isatu, age 32*

“*You know, we have these beds, there will be 10 beds, and we cultivate them every year. When you are pregnant you reduce the number of beds to 5.”—Bineh, age 27*

“*Normally I have 16 beds, when I am pregnant I only use 10 beds, and the rest I give to people to use it...for the one [baby] inside, I don't want for it to be disturbed, especially near when I am due, so I make sure to not work as much.”—Nyima, age 41*

Women shared a range of techniques for coping with the heat that they would be interested in implementing but were unable to for a variety of reasons. The primary obstacles to implementing alternative adaptive strategies were economic or logistical—all but two communities did not have electricity, limiting access to ice or other cooling devices like a fan or air conditioner. For others, the obstacle was primarily financial—even if available, they would not have funds required to implement the adaptation strategy. Some women shared social norms around dress as an obstacle to using alternative coping strategies—at home, women would often change their clothing to something lighter and looser to cool down, but social norms around dress and modesty prevented them from doing this while in the field.

“*I have thought of cold water, in my mind I think ‘If I had cold water, I would use that one” but I cannot afford it.” Aisha, age 30*.

One woman shared that she wasn't interested in trying out new methods of coping with the heat, because it might interfere with her work, and the heat was not bothersome enough to merit the adaptation.

“*…. if I had that, I can't say that I wouldn't like it, because it might stop my work – it might be a waste of time.”—Mariama, age 31*.

Women who relied on working on the farm as their only livelihood often had less ability to change or reduce the work they did during pregnancy. Some women shared that they were interested in changing the work that they did to protect themselves from heat, but faced constraints to doing so. Most often these constraints were of economic nature, and they lacked start-up capital to begin a new business.

“*I would like to do business so that I don't need to work under the sun. {what is stopping you from doing this?} I don't have the money.”—Bineh, age 27*.

“*You feel tired. Especially when you are pregnant, we are farmers, and we can't prevent ourselves from working when we are pregnant. Especially me, I am small, and my stomach is big, it is disturbing me.”—Aicha, age 30*.

### Perceptions and Understanding of Climate Change

During the interviews women were asked about any observed changes in the weather and asked to share their thoughts around the causes of climate change, as well as their perception of its present and future impacts. All women in our study shared that they have observed changes in the weather in the last 5–10 years. The changes most often mentioned were increases in heat, increase in intensity and impact of the sun, and a reduction and shortening of rainfall in the rainy season.

“*The rain has reduced. Before the water that we would get from the rainfall, we don't get the same amount now. And the sun also usually harms us now, even our crops, it destroys them.”—Mariama, age 31*.

“*When we were young, the amount of rain was more, but now it has minimized.”—Nyima, age 41*.

Many women shared that they were aware of climate change as a process, with only a few not familiar with the concept. Some associated their observed changes in weather with global climate change, but others were uncertain of the cause, or associated the changes in weather with the cutting down of trees in their community.

“*{What is causing the climate to change?} Because of the cutting down of trees. Because of the little rainfall. Because of the heat also. Because when there are not enough trees the heat is too much.”—Fatmata, age 22*

“*The generation has changed now. Because the amount of rain we used to receive was always high, but now the sun is always hot. Maybe a new generation has come now.”—Zainabou, age 40*.

Many women associated climate change with a reduction in the amount of production they were able to achieve from their agriculture, and shared that their crops have been significantly affected by the changes in weather observed over recent years.

“*What we normally farm, in the last three years, we don't gain much....when we sowed the seed, and it started to germinate, the rain stopped coming, and the seedlings dried up, so we didn't store much seed for the next rainy season.”—Fatou, age 28*

“*For the previous three years, the amount of rain we received was not like normal, at the collection points the water will not last until the harvest period, because of shortage of water.”—Isatu, age 32*

The social customs and support networks around farming were also influenced negatively by reduced water availability.

“*Usually we help each other, we used to help each other, when we finished watering we would help others. But now that the water is less we go in shifts.”—Nyima, age 40*.

Women also shared a range of insight onto the gendered impact of climate change. In their perspective, everyone is affected by climate change, but those who rely on farming as their primary source of income, and those who bear the social responsibility for providing for the family are the most affected. In most cases men bear the responsibility for providing for the household—they most often cultivate cash crops that are used for income, and if their crops fail it has significant economic and social consequences for the family. However women also bear the effects of climate change given their role as primary caregiver and responsibility for cooking.

“*The women, because we are the ones with the children at home…when you are coming to the kitchen, and you don't find anything, when you don't find the ingredients it disturbs you. If what you have [to eat] is going to be finished soon, you think about it all the time.”—Fatou, age 28*

Feelings of stress around food or income scarcity from the future effects of climate change were common among nearly all women interviewed. In nearly all cases, women discussed the close connection between the environment and their livelihood and explained that any changes in climate would have a direct influence on their ability to provide for their needs and those of their family.

“*It really affects me, since at the end of the rainy season I didn't have money up to my expectations, and there are children around me, my husband is also a farmer, and he is not working, and he doesn't know where to get money from. We have children at school, and it really affects us.”—Binta, age 31*

Many women shared some uncertainty around the impacts of climate change on their future and that of their families and their children. Often a common strategy to adapt to this change was to invest in the education of their child in the hopes that they would secure a job, or of having a family member migrate elsewhere and return remittances as a means of diversifying their income and removing their dependence on farming as a primary livelihood.

“*The only hopes I have for my children is that they work hard and become educated and to get a good job in the future. For me, I am not educated, the only thing I depend on is farming, and I don't want my children to go through that.”—Nyima, age 40*.

“*If it continues like this, it might affect me and my family. The only thing we depend on is farming, and if we cultivate crops but there is no rainfall, but don't get anything out of it, it will be very hard. If you don't have any helper or anyone sending money from abroad, it will be very hard.”—Aicha, age 30*.

One woman spoke of her husband obtaining a job nearby to supplement their income as a factor that allowed her to reduce the most physically strenuous aspects of her agricultural work, and another woman spoke of the positive economic impact of her husband who had migrated to Europe to work, and shared about how much of a struggle it was to return to being reliant only on farming upon his return.

“*Before, their father was abroad, and he would take care of us. But now he is back, and we struggle, whatever we get we use to eat.” Aisha, age 30*.

## Discussion

It is clear that all women who participated in the study experienced significant symptoms of heat illness from working outdoors during their pregnancy, with physical effects that have significant implications on their feeling of wellbeing, and reduced their overall productivity. This finding aligns with the few published studies on pregnant farm workers. Flocks et al. study of pregnant farm workers in Florida, USA, found the most common symptom of heat illness was dizziness and fainting (13). A further study among farm workers from Florida by Runkle et al. found high rates of pain or discomfort reported by pregnant farmers, but no impact on birth outcomes (28). While there are differences between these studies and the Gambian context, the women farm workers in Florida are limited by many of the same socioeconomic pressures as Gambian women farmers in that their income is contingent on their productivity on the farm, and workers may push themselves beyond their heat tolerance to avoid compromising their livelihood. Understanding whether and how women are able to cope with heat stress through developing mechanisms to minimize the impact of heat exposure is key to protecting the health of women exposed to heat stress. Our study's intersectionality lens makes an important contribution to furthering this understanding.

### Coping Mechanisms Are Shaped by Intersectionalities

A key finding of this study is that layered identities, experiences, and household power structures shaped the extent to which women who participated in the study were able to prevent and reduce the effects of heat exposure during their work whilst pregnant. A predominantly patriarchal society with rigid social norms around gender limited their access to resources, determined the economic activities and opportunities available to them (in turn shaping their economic mobility) and limited their ability to take adaptive action to mitigate their risk of heat stress, including their agency in decision-making surrounding their reproductive health.

The most common adaptive techniques included resting in the shade while working, completing their work in multiple shorter time increments, taking medication or herbal medicine to reduce symptoms like headache, and using water to cool down. A common adaptation was reducing the amount of area they cultivate while pregnant—sometimes this was a choice made to protect themselves and their fetus, and in other situations women were incapable of working to the same extent because the physical effects of pregnancy were so severe. While studies have shown that self-pacing of workload and taking regular breaks from heat exposure during the day can be an important means of reducing the risk of occupational heat stress among outdoor workers (29), income lost was not often replaced by other means, and this often had significant economic implications for the woman and her family. In addition, women's ability to self-pace their work while in the field was limited in some cases by collective work where social norms expect all members to contribute equally, or by an overwhelming workload that left limited time for rest. Given recent evidence on the importance of self-pacing for limiting the negative effects of heat stress and increasing worker productivity (30), further research into these dynamics, as well as into interventions such as education around occupational heat adaptation and productivity for women and their communities, could serve to better inform health promotion strategies surrounding worker-related heat stress in pregnancy.

Several intersecting identities beyond gender had important implications on women's ability to participate to implement coping strategies to mitigate heat stress during their pregnancy. In nearly all cases, the woman's identity as a mother brought on a significant burden of socially obligatory unpaid care work that limited their available time for rest or leisure while pregnant. Roles surrounding motherhood (childcare, cleaning, cooking) are firm, and there is little space to negotiate household care activities with their spouse.

Age was an important factor that often afforded privileges not available to younger women—older women had a higher level of agency in deciding what kind of work to carry out, and more space to negotiate decisions with their husband. Age also often brought more economic stability and social support, and older women often had more social connections to leverage protectively—older children, especially female children, were often able to help them in their work in the field whilst pregnant, or with their domestic responsibilities, though they were unable to when studying, or if they had traveled for schooling or economic activities in the city. Socioeconomic pressures shaped women's ability to adapt their work, which in turn translated directly to increased susceptibility to heat stress by needing to work even whilst suffering the effects of heat, and/or more acute implications on their quality of life if obliged to reduce their workload (and resulting earnings) while pregnant. Marriage status, such as being in a polygamous household, often limited or expanded the resources available to draw upon to adapt work during pregnancy and beyond. Some nuance surrounding this dynamic was outlined in the experience shared by one woman who was able to split household duties with her co-wife, enabling more time for rest during her pregnancy.

Life events such as migration to a new community was also an important factor limiting or enhancing use of coping strategies—having an identity as an “outsider” without blood relatives also shaped the social space within which women felt able to ask for support with their work. Strong familial social support systems nearby were among the most important factors that women rely on to adapt their work and reduce their exposure to heat during their pregnancy. The strongest social supporters were mothers and sisters, and residing in the same community as their mother and sisters often enabled women to take more time to rest during their pregnancy, and reduced their stress around the risk of not working. Women who left their families and community of origin often had fewer social connections to draw upon to support them in their work, and therefore less ability to implement measures to protect themselves from the heat. This raises important questions about the critical role of social networks in climate change adaptation, since social relationships are often fragmented in situations of climate-induced migration and conflict.

### Addressing Climate Change Challenges Faced by Women

In our study, climate change was universally recognized as an important challenge for women. Effects of changes in rainfall were most significant as they reduced the amount of crops harvested, with significant implications for their income and livelihood. Effects of climate change were perceived to impact everybody, but particularly those who rely completely on their harvest for food, and those who bear the social and economic responsibility for the primary household income (cash crops, most often men), or for immediately providing for household needs. Women are responsible for cooking and providing food for the household on a daily basis, as well as childbearing and childcare, and often face feelings of stress toward the changes in climate which make their work harder. While income diversification was a common theme that emerged in the data as a means of enhancing resilience to climate change, it was usually limited to children or the male head of households obtaining additional work. Economic opportunities for women are limited by a range of factors, not least of which is a lack of reproductive autonomy that often obligates women to bear the physical burden of bearing and caring for many closely spaced children. Some women recognized this dynamic by emphasizing that with more space between children, they and their families would be healthier and better able to cope with the demands of work.

At the structural level, women are often missing from research, policies, and decision-making surrounding climate change, whilst their bodies, livelihoods, families, and communities are disproportionately affected by it. This is a trend reflected in climate change discourse, academia, and public policy globally, including The Gambia (8, 31). Women who participated in this study demonstrated high awareness of climate change, and offered important insights into potential values, priorities, and mechanisms to enable effective adaptation. There is an important opportunity to make space for women's leadership within climate change discourse, policy-making spaces, and design of development programming in The Gambia to enable better climate adaptation.

### Limitations

While the study attempted to recruit pregnant women with a range of intersecting identities, the sample had a smaller than average number of young mothers (between 18 and 25), and the average age of participants in the study was higher than for pregnant women in the communities (32 vs. 28 years). Additionally we did not include pregnant women who were single or from groups that are known to be particularly marginalized, including teenage mothers. Though the sample was reflective of the demography of many women in West Kiang, future studies would benefit from deliberate recruitment of women from marginalized female population groups. If needed the geographic area of the study could be expanded and used as an opportunity for triangulation of findings. Similarly, the time for data collection could be extended to enable women to share more immediate experiences of heat and pregnancy in different seasons throughout the year. While women were asked about the differences in their physical experience when working in the heat while pregnant compared to when not pregnant, in future research, interviewing women who have not been pregnant or who have not recently been pregnant on their experience working during the heat would allow greater attribution of the experiences shared to the effects of pregnancy. In addition, there is currently a disconnect between environmental epidemiological studies that demonstrate the association of heat exposure on increased risk of preterm births, low birth weight and stillbirth and the individual lived experiences of women and their pregnancy outcomes. Future work could focus on perception of worsening birth outcomes in populations exposed to extreme heat/climate shocks.

While the design of the study allowed the research team to gather initial insights into individual experiences in regards to heat and climate change, having a non-local researcher part of the interview process introduced a risk of power hierarchy and potential source of courtesy bias. Though having a non-local researcher with experience in conducting interviews for qualitative research part of the interview process was valuable to make data gathering, processing, and analysis more efficient for the research team, it is possible that women did not feel comfortable sharing openly with someone from outside their community due to a perceived power differential. The research team attempted to balance this risk of courtesy bias by having a researcher who is known to the community introduce the study and describe the identities of the research team in advance of the interviews and during the consent process, allowing women a safe space to raise questions or concerns before committing to participate. At the interview, interviewers took extra time to get acquainted with the women, and used a culturally sensitive, consent-based style of interviewing. For this reason, this study is only an initial, limited, insight into the experiences of Gambian women farmers in regards to climate change and working in the heat whilst pregnant, and further work is needed to more completely understand and center their perspective on these topics. In future, using peer-based interviewing or focus group methods could help to reduce the risk of power imbalances between researcher-participant identities, deepen analysis surrounding the shared experience of working as a farmer in the heat, gather consensus around recurring themes and findings, and create spaces for collective discussion and knowledge-generation surrounding climate adaptation strategies.

## Conclusion

In the context of the reality that there is a large global female workforce who perform manual labor outdoors during their pregnancy, this study highlights the need to address the potential harms of increasing heat exposure with climate change. Our findings reveal many intersecting social and economic factors that shape the space within which women can make decisions and take adaptive action to reduce the impact of heat during their pregnancy. To improve the health of pregnant working women exposed to heat, these intersectionalities must be taken into account when supporting women to adapt their working practices and cope with heat stress.

## Data Availability Statement

The raw data supporting the conclusions of this article will be made available by the authors, without undue reservation.

## Ethics Statement

The study was approved by MRC/Government of Gambia Joint Ethics Committee and LSHTM Ethics Committee. The patients/participants provided their written informed consent to participate in this study. Written informed consent was obtained from the individual for the publication of any potentially identifiable images or data included in this article.

## Author Contributions

SS: interviewing, data interpretation, analysis, manuscript writing, and revision. TS: community liaison, interviewing, data interpretation, and manuscript edit. KW and SM: research design, protocol development, and manuscript edit. AB: research conceptualization, research design, protocol development, manuscript writing, and edit. All authors contributed to the article and approved the submitted version.

## Funding

This project was funded by a Wellcome Trust Global Health PhD Fellowship awarded to AB (216336/Z/19/Z).

## Conflict of Interest

The authors declare that the research was conducted in the absence of any commercial or financial relationships that could be construed as a potential conflict of interest.

## Publisher's Note

All claims expressed in this article are solely those of the authors and do not necessarily represent those of their affiliated organizations, or those of the publisher, the editors and the reviewers. Any product that may be evaluated in this article, or claim that may be made by its manufacturer, is not guaranteed or endorsed by the publisher.
