# Supplementary material for: The Challenges of Working in the Heat Whilst Pregnant: Insights From Gambian Women Farmers in the Face of Climate Change
Source: Front Public Health. 2022 Feb 10;10:785254. doi: 10.3389/fpubh.2022.785254 (PMC8883819; doi:10.3389/fpubh.2022.785254)
Supplement: Supplementary file 1 [file Data_Sheet_1.docx]

**Topic guide: Climate change and its health impacts on rural Gambian female farmers; a qualitative study of local understanding and adaptations focused on pregnancy (CHIQ)**

**Version 3.0**

**25/03/2021**

I want to thank you for taking the time to meet with me today. My name is___________________ and I would like to talk to you about your experiences as a farmer and (becoming) mother, especially with regard to heat.

The interview should take about an hour and a half. I would like to record the session because I don’t want to miss any of your comments. Are you happy for this to happen? No-one outside the project team will have access to the recording and after the data have been transcribed and analysed the recordings will be destroyed. I will also be taking some notes during the session to remind myself of important thoughts and ideas.

All responses will be kept confidential. This means that your interview responses will only be shared with my supervisors and we will ensure that any information we include in our report does not identify you as the respondent. You don’t have to talk about anything you don’t want to share and you may end the interview at any time.

Are there any questions about what I have just explained? Are you willing to participate in this interview?

| Warm-up questions:  How old are you? / When were you born?  Have you always lived in this place?  Tell me about your family.  {depending on whether she’s pregnant or has just delivered}: How many children do you have? Is this the first time you are pregnant? How are you feeling with this pregnancy?  **Working habits & pregnancy**   1. Please describe your “workplace” [“farm/garden”] and the type of work that you do.   [probe] Can you tell me about any other people you work with? Who helps you with your work?   1. When did you start working outside regularly? 2. Please describe if and how your working habits changed when you became pregnant. 3. What is the weather usually like when you are working?  - Are there changes over the year? - Can you describe the weather over the course of a working day from morning to evening? - Can you describe what working outside is like usually when it is hot? - Can you describe what working outside is like when it is very hot, and you are pregnant versus when you are not pregnant?  1. {depending on whether she already has had children or not}: How was working outside when it was hot during your other pregnancies? 2. Can you describe if the heat impacts you and the baby?  - [probe, if speaking in hypothetical] Can you tell me about a time that you experienced the impacts that you described yourself?   What do you currently do to protect yourself and the baby against the heat during work?   1. According to you, what could be important things to do when it is very hot to protect yourself (and the baby)?  - Probe: Can you please describe how could you adapt your work to reduce the impact of the heat?  1. Do you feel you are able to take these measures?  - Probe: What is stopping you from taking these measures? - Probe: What would you need in order to use these measures?  1. What would happen if you could not go to work?   **Perceptions of climate change:**   1. From your experience, has the weather changed over the last years?  - {if yes}: How? Why do you think this is happening? - {if yes}: In your opinion, have these changes affected everyone equally? - {if yes}: What impacts does the changing weather have on you as a farmer? - **{if no}:** What does climate change mean to you? Do you think it will have any impacts? Please describe from your understanding of which impacts, where, and on whom.  1. According to you how will the weather continue to change in the future? 2. Describe how you think your life and that of your children will be affected by these changes in future years. 3. Can you describe any hopes you might have for your children’s future? 4. Can you describe any worries you might have for your children’s future?   **Reproductive decision-making:**   1. Can you describe how you usually decide to become pregnant?  - What factors influence whether you decide to become pregnant?  1. How do you feel about having this pregnancy/baby?  - Why did you want to have another baby at this time?  1. How do you feel about family planning/contraception?  - What factors would make you want to use family planning or contraception? - Could you get access to contraception if you wanted to use it? Please describe how and where. - Can you describe any barriers you feel you would face to accessing family planning?   **Non-leading prompts for interviewer:**  That sounds very interesting, can you tell me more about this?  Could you expand more on …  You mentioned before ... can you explain that in more detail?  How did you feel when … happened?  What were you thinking about … ? |
| --- |
